# Out-of-pocket expenditure for home and facility-based delivery among rural women in Zambia: a mixed-methods, cross-sectional study

**DOI:** 10.2147/IJWH.S214081

**Published:** 2019-08-01

**Authors:** Jeanette L Kaiser, Kathleen L McGlasson, Peter C Rockers, Rachel M Fong, Thandiwe Ngoma, Davidson H Hamer, Taryn Vian, Godfrey Biemba, Jody R Lori, Nancy A Scott

**Affiliations:** 1 Department of Global Health, Boston University School of Public Health, Boston, MA, USA; 2 Department of Research, Right to Care Zambia, Lusaka, Zambia; 3 Section of Infectious Diseases, Department of Medicine, Boston Medical Center, Boston, MA, USA; 4 School of Nursing and Health Professions, University of San Francisco, San Francisco, CA, USA; 5 National Health Research Authority, Pediatric Centre of Excellence, Lusaka, Zambia; 6 Department of Research, Office of Global Affairs and Pan American Health Organization/ World Health Organization Collaborating Center, University of Michigan School of Nursing, Ann Arbor, MI, USA

**Keywords:** cost, skilled birth attendance, obstetric care, maternal health, social determinants of health, sub-Saharan Africa

## Abstract

**Purpose:**

Out-of-pocket expenses associated with facility-based deliveries are a well-known barrier to health care access. However, there is extremely limited contemporary information on delivery-related household out-of-pocket expenditure in sub-Saharan Africa. We assess the financial burden of delivery for the most remote Zambian women and compare differences between delivery locations (primary health center, hospital, or home).

**Methods:**

We conducted household surveys and in-depth interviews among randomly selected remote Zambian women who delivered a baby within the last 13 months. Women reported expenditures for their most-recent delivery for delivery supplies, transportation, and baby clothes, among others. Expenditures were converted to US dollars for analysis.

**Results:**

Of 2280 women sampled, 2223 (97.5%) reported spending money on their delivery. Nearly all respondents in the sample (95.9%) spent money on baby clothes/blanket, while over 80% purchased delivery supplies such as disinfectant or cord clamps, and a third spent on transportation. Women reported spending a mean of USD28.76 on their delivery, with baby clothes/blanket (USD21.46) being the main expenditure and delivery supplies (USD3.81) making up much of the remainder. Compared to women who delivered at home, women who delivered at a primary health center spent nearly USD4 (p<0.001) more for their delivery, while women who delivered at a level 1 or level 2 hospital spent over USD7.50 (p<0.001) more for delivery.

**Conclusion:**

These expenses account for approximately one third of the monthly household income of the poorest Zambian households. While the abolition of user fees has reduced the direct costs of delivering at a health facility for the poorest members of society, remote Zambian women still face high out-of-pocket expenses in the form of delivery supplies that facilities should provide as well as unofficial policies/norms requiring women to bring new baby clothes/blanket to a facility-based delivery. Future programs that target these expenses may increase access to facility-based delivery.

## Plain language summary

Costs of delivering at a health facility make it challenging for rural, poor women in sub-Saharan Africa to access maternity care. We analyzed the costs for delivery of the most remote women in Zambia and compared differences between women based on where they delivered their most recent baby (clinic, hospital, or home). We conducted household surveys with 2280 randomly selected remote Zambian women who had delivered a baby within the previous year. Women reported what they spent for delivery supplies, transportation, baby clothes, diagnostic tests, and medications, among others. Approximately 98% of women reported spending money on their delivery. Nearly all women (96%) spent money on baby clothes/blanket, while over 80% purchased delivery supplies such as disinfectant or cord clamps, and about a third (36%) paid for transportation. On average, women reported spending USD29 on their delivery, with baby clothes/blanket (USD21) being the main cost and delivery supplies (USD4) making up much of the remainder. Women who delivered at a clinic or hospital spent about USD4 and USD8 more, respectively, than women who delivered at home. The poorest Zambian households spend approximately one third of their monthly household income on delivery. While the outlawing of health center fees for maternity care in Zambia has reduced the direct costs of delivering at a health facility for the poorest members of society, remote Zambian women still face high delivery costs. Future programs that try to reduce these costs may help women access health facilities for delivery.

## Introduction

Although maternal deaths are largely preventable,^1^ maternal mortality and other adverse birth outcomes, including early neonatal mortality, remain high in many low and middle-income countries,^2^–^5^ such as Zambia.^6^ The World Health Organization recommends women deliver at capable health centers with trained health care providers, known as skilled birth attendants, to reduce adverse outcomes.^7^ However, barriers that hinder women from delivering at health facilities exist at many levels including low country-level spending on health (societal level), limited availability of quality health services (community level); long distances or other physical barriers to reaching health facilities and limited access to transport (community level); insufficient social/familial support (interpersonal level); limited household wealth and low maternal education (individual levels), among others.^8^–^11^ These barriers interact in complex ways that can limit the likelihood of women delivering with skilled birth attendants.

Delivery-associated costs that must be paid out-of-pocket (OOP) are a well-documented barrier to facility-based delivery.^8^–^10^,^12^,^13^ Women in low-resource settings often face high OOP costs in the form of facility user fees, tests, medicines, delivery supplies (including disinfectant, gloves, cord clamps, and a plastic sheet), and transport to the health facility.^8^–^10^,^12^–^14^ OOP expenditure is a particular burden on rural and lower socioeconomic status women.^10^,^14^ In response, several countries have abolished user fees to increase access to health services.^15^–^17^ While Zambia’s abolition of user fees for primary health services in 2006 likely increased access to general primary health services in rural districts,^18^,^19^ the same effect has not been shown regarding access to facility delivery.^20^ Many rural Zambian women still find it difficult to pay delivery-associated expenses, which serves as a barrier to accessing facility-based delivery.^21^–^23^

In the National Health Strategic Plan for 2017–2021, the Zambian government set a target to decrease the maternal mortality ratio from 398 to 162 maternal deaths per 100,000 live births by 2021, largely by increasing access to skilled birth attendance in health centers with sufficient trained staff and equipment to provide emergency obstetric and neonatal care.^24^ Understanding existing barriers is essential to improving access to skilled birth attendance. However, relatively little is known about the financial burden of delivery in Zambia, particularly among the most rural, and most socioeconomically disadvantaged women. This paper quantifies and qualitatively explores this financial burden, including how much women are spending for delivery, what they are spending on, and how those expenditures compare among women who delivered at primary health centers, hospitals, and at home.

## Methods

### Study setting

This analysis was conducted using data collected as part of the baseline evaluation of a maternity waiting homes project in rural Zambia.^25^ Forty rural health centers, known henceforth as primary health centers for this article as it is a more internationally recognized term, were chosen from among those that met the following eligibility criteria: travel to a referral hospital within two hours; capacity of health staff to perform at least five out of seven basic emergency obstetric and neonatal care (BEmONC) signal functions;^26^ and volume of deliveries ≥150 per year. The study was conducted in seven rural districts: Nyimba and Lundazi in Eastern Province; Mansa and Chembe in Luapula Province; and Choma, Pemba, and Kalomo in Southern Province. The study districts are primarily rural, ranging from 67% of the population in Mansa/Chembe District (administratively combined for the 2010 Census) to 95% in Lundazi, with pockets of peri-urban centers.^27^ Each district have one or more hospitals, either Levels 1 or 2, excluding Chembe which refers to the neighboring Mansa District Hospital, and an average of 22 primary health centers, ranging from five in Chembe to 33 in Choma/Pemba (administratively combined in The 2012 List of Health Facilities in Zambia).^28^ All of the hospitals and nearly all of the primary health centers in these seven districts are considered delivery sites, though their capacity to perform BEmONC functions varies.^28^ More details on the Zambian health system and the levels of care can be found elsewhere.^28^

### Study design and data collection

A household survey was conducted among women who delivered a baby in the 13 months prior to data collection (April-May 2016) and lived more than 10 kilometers (km) from their assigned primary health center. Women were chosen through a multi-level random sampling process. Within the catchment area of 40 study sites, we randomly selected villages with centers more than 10km (rounding up from 9.5 km) away from their designated health center. We randomly selected households to approach from all eligible households in the village, and then randomly selected a woman from the household if more than one woman was eligible. A subsample of 10% was randomly selected to participate in an in-depth interview (IDI) immediately following the survey to gain deeper insight into community and personal perspectives on delivery location, maternity waiting homes, and delivery-associated expeditures.

The household survey took approximately 60 minutes to complete and captured demographic information, including age, education, martial status, household assests, number of previous pregnancies (gravida) and births (parity), among other variables, as well as information around the most recent pregnancy experience, from antenatal through the postpartum period. The survey also included questions about expenditures associated with delivery. In reference to their most recent delivery, we asked women to estimate how much they spent in preparation for delivery (ie on supplies and baby clothes/a baby blanket); on the journey for delivery (ie on roundtrip transportation, accommodation while awaiting delivery); and at the time of delivery or immediately afterward (ie on provider fees, medicines, diagnostic tests, informal payments, tips, and in-kind contributions). All expenditures were reported in the local currency, Zambian kwacha (ZMW).

The IDIs took approximately 25 additional minutes to complete. The interview guide included a total of 20 questions on community and personal delivery practices, preparedness and costs, and perspectives on maternity waiting homes. Six questions asked respondents how they had prepared for their last delivery and what expenses they incurred, eliciting detail on what they spent money on, how much was spent, and how those expenses would have differed if they had delivered in a different location. The household surveys and IDIs were conducted in a private location of the respondent’s choosing, usually just outside their household. Additional information on the setting and sampling methods for the evaluation is available in the published study protocol (Clinicaltrials.gov: NCT 02620436).^25^

### Data management & analysis

Quantitative data were captured using SurveyCTO Collect software (Dobility, Inc, Cambridge, MA) installed on encrypted tablets. Data were cleaned and analyzed using SAS version 9.3 (SAS Institute, Cary, NC). The categories of items that women could have spent money on for delivery (expenditure categories) were collapsed into the following: (1) total expenditure; (2) baby clothes/baby blanket; (3) delivery supplies (such as disinfectant, gloves, cord clamps, a plastic sheet, a razor blade, a bucket, etc); (4) transport to and from the delivery location; (5) accommodation while awaiting delivery; (6) facility fees, including provider fees, medicines, and diagnostic tests; and (7) other costs, including informal payments, tips, and anything else the respondents included that did not fit into one of the prior categories. The following categorical variables were created for this analysis: whether the woman reported spending anything on delivery (yes/no), and the season of the woman’s delivery (rainy/dry).

Women in this analysis delivered at primary health centers, first or second level hospitals, or their own or another’s home. Responses to the question in the household survey asking about delivery location aggregated primary health centers and health posts as a delivery location. Not all health posts are considered delivery centers as they often lack full-time staff or skilled birth attendants. However, based on the name of the facility provided, very few women delivered at a health post among the final sample. We have excluded from the analysis the 77 (3.2%) women who delivered on the way to a facility because their spending patterns do not reflect intentional behavior. We have excluded six (0.3%) women where delivery location was unknown and one (0.04%) where the woman lived too close to her assigned health center. We have also excluded 17 (0.7%) women whose spending behavior was considered extreme outliers (ie spent more than USD100 in any category besides total expenditure). These records may have resulted from incorrect data entry, with additional zero’s added, or from reporting in Malawian Kwacha (MWK) instead of Zambian Kwacha, which has an exchange rate of approximately MWK50 to ZMW1.

We combined the districts of Choma and Pemba as well as Mansa and Chembe, respectively, as each pair was previously administratively combined and the population of each pair is demographically and behaviorally similar. Pemba and Chembe districts each have two study sites included in this cross-sectional study.

Descriptive statistics were calculated for the subset of women who reported any expenditure data and compared to the total sample using chi-squared tests of homogeneity and two-sample *t*-tests. A household asset index was constructed based on responses to a series of household asset questions taken from the 2014 Zambian Demographic and Health Survey.^6^ Wealth quartiles were constructed based on the household asset index. We calculated the proportion of women who reported spending anything on delivery and in each of the expenditure categories. We then calculated the mean and standard deviation for the reported expenditure of the total sample, which included individuals who did not report any expenditure. We include the median and interquartile range (IQR) when discussing amount spent for the subset of women who reported any expenditure within each category. We display box plots for all expenditure categories by delivery location for the main four categories of expenditure (ie total expenditure, baby clothes/baby blanket, delivery supplies, and transportation). Expenditure data were converted to US dollars (USD) using the average ZMW to USD exchange rates from March 2015 to May 2016.^29^

We employed a two-part modelling approach to account for the skewed distribution of the expenditure data, per the method recommended by Deb and Norton (2018).^30^ First, we fit a series of logistic regression models to predict the odds of any expenditure overall and within the top three categories of expenditure (ie baby clothes/baby blanket, delivery supplies, and transportation). Second, we fit a series of log transformed generalized linear models excluding data from households that reported no expenditure, to explore associations between select covariates and level of expenditure.^30^ Based on an earlier analysis which used these data to assess predictors of home delivery,^23^ the models for this analysis included the following covariates: age, education level, marital status, wealth quartile, district, prime gravida (first pregnancy), antenatal care (ANC) attendance (four or more visits), whether a woman saved for delivery, season of delivery, and distance from assigned health center. Mode of transport was not included in the models because it was only collected for facility-based deliveries. The largest category was used as the reference for each covariate in the models of total expenditure.^31^ Finally, we fit a series of models to explore differences in expenditure by delivery location. Home delivery was used as the reference category for the two-part model on expenditure by delivery location.^31^ Observations within each sub-category that reported an expenditure of more than USD100 were excluded from the figures and models as they were identified as substantial outliers, though total expenditures greater than USD100 were retained. Data were considered statistically significant at p≤0.05.

IDIs were audio recorded, translated into English using individuals fluent in the relevant local languages, and transcribed verbatim into Microsoft^®^ Word. The IDIs were coded and analyzed in NVivo v11 © (QSR International, Doncaster, Australia). The main codes were identified a priori based on the interview guide and sub-codes were created and refined as they emerged from the IDIs. We conducted a content analysis of emerging themes surrounding delivery expenditure and compared the results by district and by delivery location. We have included the US dollar conversion for any Zambian kwacha amounts mentioned in the illustrative quotes using the average exchange rate from March 2015 to May 2016.^29^

We triangulated the quantitative and qualitative data and present the results together. For each finding, we usually first present the quantitative result then the associated qualitative result for deeper understanding. While in most cases the qualitative findings corroborate the quanitative findings, we note in the results section where the related quantitative and qualitative findings are inconsistent.

## Results

### Sample characteristics

A sample of 2280 women were included in this analysis; 232 (10.2%) also completed an IDI. Among the total sample, 2223 (97.5%) women reported spending a non-zero amount on delivery. Households in the study were generally poor, with most having no improved toilets (90.0%), no electricity (99.7%) and earth or sand floors (88.2%) (Table 1). Households were in villages located a median of 12.7 km from their assigned primary health centers. Respondents had a median age of 24 years, were married or cohabitating (87.8%), and had at least some primary education (84.9%). Just over 21% of women were reporting on their first pregnancy; about 59% had attended the recommended four or more ANC visits. Approximately 84% of women delivered their last child at a primary health center or at a hospital, while about 16% delivered at home. There were few demographic differences between the total sample and women who reported any expenditure.

### Expenditure among the total sample

**Figure 1:**
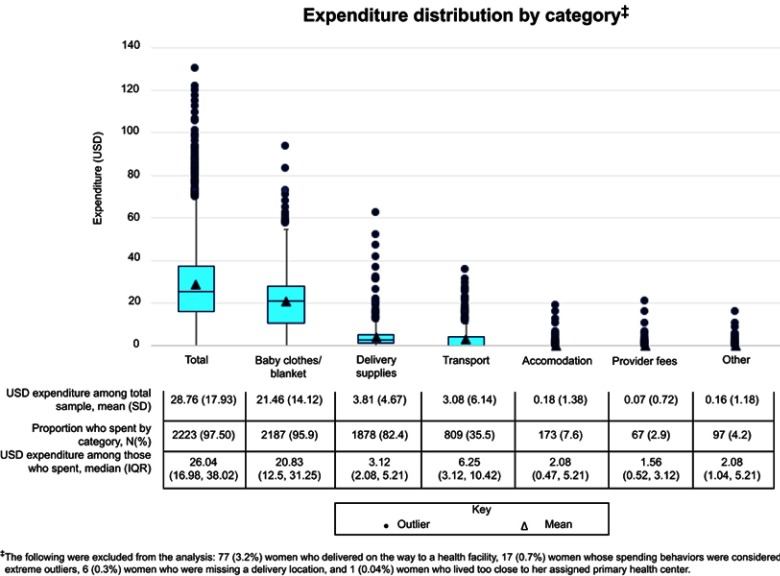
Distribution of expenditure by total expenditure and all expenditure sub-categories (N=2280).

Among the final total sample of 2280 women, respondents spent a mean of USD28.76 on their deliveries. Nearly all respondents in the total sample spent money on baby clothes/blanket (95.9%) spending a mean of USD21.46 (Figure 1). Women who participated in the IDIs commonly discussed baby clothes/blanket as a major expense regardless of their delivery location, explaining that the health facility often specifically requires new clothes be brought for a delivery:

“I bought all those things that we were told at the clinic like baby blanket, clothes, gloves, bleach. The thing which is most expensive, when you deliver from the clinic, is buying a baby blanket.” – Woman, Kalomo District (delivered at primary health center)

“When you deliver a child from the clinic, they don’t allow you to use the clothes that are old. They want new clothes.” – Woman, Lundazi District (delivered at primary health center)

“I think it’s the baby clothes which are very expensive when you deliver from the health center. Even if you deliver from home, baby clothes are still expensive to buy.” – Woman, Kalomo District (delivered at a primary health center)

“I think the baby blanket was costly. It is the most expensive item that I bought.” – Woman, Lundazi district (delivered at home)

Over 80% of women reported spending on delivery supplies, such as disinfectant, gloves, and cord clamps. Among those who spent something on supplies, the mean expenditure was USD3.81. During IDIs, women frequently discussed delivery supplies as a requirement for health facility deliveries, but also necessary for home deliveries:

“If you don’t manage to get what they require you to buy, the health staff charge for gloves and bleach about 50 kwacha (USD5.21).” – Woman, Mansa District (delivered at a primary health center)

“I bought everything – bleach, plastic, a dish, umbilical cord clamps, gloves, napkins, a baby blanket and a brand new chitenge wrapper (fabric to cover the delivery bed and for the mother to wear). Everything.” – Woman, Kalomo District (delivered at home)

Slightly more than a third of respondents spent money on transportation. Among those who spent something, the mean expenditure was USD6.25. Evidence from the IDIs suggests that spending on transportation was influenced by the time of day a woman was travelling, by how quickly the woman needed to get to the clinic or hospital, and by the types of transport available to her at the time. When labor began at night, women reported, transport options were limited and costlier. Similarly, women stated that a car/taxi/bus is faster though more costly than an ox cart. IDI respondents frequently discussed the cost of transportation and the nuances involved in this cost:

“We used the transport money to go to the hospital. If it happens at night, you use 180 or 190 kwacha (USD18.75-USD19.79) but if you go during the day to the hospital, you used 100 kwacha (USD10.42) for booking.” – Woman, Nyimba District (delivered at a primary health center)

“It depends on how you negotiate with the owner of the ox cart, some people will charge you 20 kwacha (USD2.08), some maybe you negotiate for 10 kwacha (USD1.04). But if you have to book a vehicle you spend 150 kwacha (USD15.63).” – Woman, Choma District (delivered at home)

Fewer than 8% of respondents reported spending on accommodation; under 3% on facility/provider fees, medicines, or diagnostic tests; and under 5% on informal payments and tips (Figure 1). Due to the low proportion of reported spending on accommodation, fees/medicines/tests, and other items, the mean amount spent on each of these categories among the total sample was less than USD1. When women who reported no spending are excluded, the median among those who spent on each category was USD2.08, USD1.56, and USD2.08 for accommodation, fees/medicines/tests, and other items, respectively. For the categories where a high proportion of the respondents reported spending (baby clothes/blanket and delivery supplies), the difference between the population mean expenditure and the median expenditure among spenders was minimal, with the population mean being higher likely due to the lack of women spending zero amounts for these categories and the large distribution of spending above the interquartile range.

### Expenditure by delivery location among the total sample

**Figure 2:**
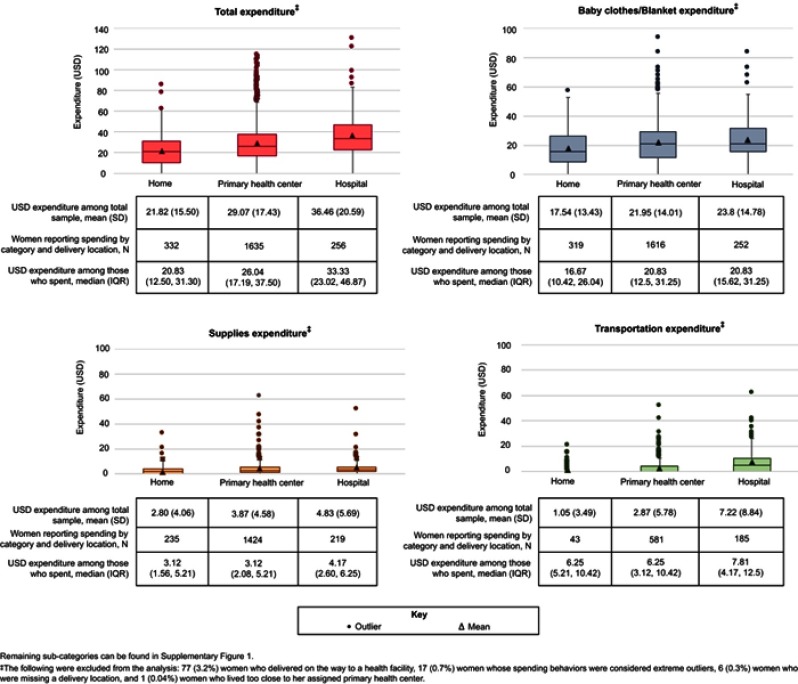
Distribution of expenditure by delivery location for total expenditure and top three sub-categories.

Mean total expenditure was higher among women who delivered at a hospital (USD36.46) or at a primary health facility (USD29.07) compared to women who delivered at home (USD21.82; Figure 2). Baby clothes remained the bulk of the expenditures for all women regardless of their delivery location, while delivery supplies remained a small but persistent expense, ranging from USD2.80 for a home delivery to USD4.83 for a hospital-based delivery.

Among women who spent on transportation, those delivering at a hospital spent the most, (median USD7.81), while those delivering at home spent the least (USD6.25; Figure 2 and explained further below). For women who delivered at either a primary health center or a hospital, expenditure on transport varied by method of transport used (Table 2). Only a third of women who delivered at a primary health center spent on transportation, while nearly three quarters of hospital deliveries did. Median amounts spent were not substantially different for the three primary methods of transport (walking, bicycle, and car/taxi/bus) between the delivery locations. Over three-quarters of women who spent on transportation and delivered at a primary health center or hospital used a car/taxi/bus as their primary method of transportation spenting a median of USD6.25 and USD7.81, respectively, greater than the modes of transport among the total sample who delivered at these locations.

**Table 1:** Characteristics of recently delivered women living ≥10km from their assigned primary health center for the total sample and only those who spent money on delivery^‡^

		Total sample (n=2280)		Spent money on delivery (n=2223)		p-value
N	%	N	%
**Household-level characteristics**						
Non-improved water source		1274	55.9	1239	55.8	
Non-improved toilet		2051	90.0	1996	89.8	*
No electricity		2270	99.7	2213	99.7	*
House has earth or sand floors		2010	88.2	1956	88.0	*
Charcoal or wood cooking fuel		2271	99.6	2214	99.6	*
Total household members	Median (IQR)	6.0 (4.0, 9.0)		6.0 (4.0, 9.0)		
Wealth index	1 (lowest)	519	24.4	498	24.0	
	2	547	25.7	535	25.8	
	3	536	25.2	523	25.2	
4 (highest)	524	24.6	518	25.0	
Distance from village center to health facility (km)	Median (IQR)	12.7 (10.9, 16.2)		12.8 (10.9, 16.2)		
9.5–10	290	12.7	281	12.7	
	10–14.9	674	29.6	655	29.5	
	15–19.9	599	26.3	586	26.4	
	20–24.9	511	22.4	502	22.6	
	25+	203	8.9	196	8.8	
District	Kalomo	380	16.7	364	16.4	
	Choma/Pemba	556	24.4	543	24.4	
	Lundazi	572	25.1	564	25.4	
	Nyimba	213	9.3	206	9.3	
Mansa/Chembe	559	24.5	546	24.6	
**Woman-level characteristics**						
Age, years	Median (IQR)	24.0 (20.0, 31.0)		24.0 (20.0, 31.0)		
	15–19	416	18.3	407	18.4	
	20–24	732	32.2	713	32.2	
	25–29	432	19.0	422	19.0	
	30–34	359	15.8	348	15.7	
	35+	332	14.6	326	14.7	
Education	None	344	15.1	340	15.3	
	Any primary	1385	60.9	1345	60.7	
	More than primary	545	24.0	532	24.0
Marital Status	Never married	123	5.4	117	5.3	
	Divorced/separated or widowed	154	6.8	146	6.6	
Married/cohabitating	1999	87.8	1956	88.1	
Gravida	Median (IQR)	3.0 (2.0, 6.0)		3.0 (2.0, 6.0)		
Parity	Median (IQR)	3.0 (2.0, 5.0)		3.0 (2.0, 5.0)		
Primigravida (first pregnancy)	No	1788	78.5	1743	78.4	
Yes	491	21.5	479	21.6	
Four or more ANC visits	No	941	41.3	902	40.6	*
Yes	1339	58.7	1321	59.4
Delivery location	Home	361	15.8	332	14.9	*
	Primary health center	1659	72.8	1635	73.5	
Hospital	260	11.4	256	11.5
Saved for delivery	No/don’t know	42	17.6	363	16.3	*
	Yes	1878	82.4	1860	83.7
Intended delivery location	Home	20	0.9	18	0.8	*
	Primary health center	2107	92.5	2055	92.5	
	Hospital	150	6.6	149	6.7
Went to health facility 24 hrs after delivery^a^		94	25.8	93	27.8	*
Mode of transportation to delivery^b^	Walking	432	22.6	427	22.6	*
Bicycle	589	30.8	575	30.5		
	Ox cart/wheelbarrow	151	7.9	147	7.8	
	Car/Taxi/Bus	617	32.2	613	32.5	
	Motorcycle	102	5.3	102	5.4	
	Ambulance	24	1.3	23	1.2

**Notes:** ^‡^The following were excluded from the analysis: 77 (3.2%) women who delivered on the way to a health facility, 17 (0.7%) women whose spending behaviors were considered extreme outliers, 6 (0.3%) women who were missing a delivery location, and 1 (0.04%) woman who lived too close to her assigned primary health center. *Chi-square test statistically significant at  p=0.05. ^a^Only asked of those who delivered at home. ^b^Only asked of those who delivered at a primary health center or hospital.

**Table 2:** Primary transport method used and amount of expenditure on transport among women with a facility-based delivery^‡^

Primary Transport Method Used	Primary health center N=1656				Hospital N=260			
	Among total sample of primary health center deliveries		Among primary health center deliveries who spent on transportation		Among total sample of hospital deliveries		Among hospital deliveries who spent on transportation	
Transport method used N (%)	Expenditure amount (USD) Mean (SD)	Transport method used N (%)	Expenditure amount (USD) Median (IQR)	Transport method used N (%)	Expenditure amount (USD) Mean (SD)	Transport method used N (%)	Expenditure amount (USD) Median (IQR)
Walking	402 (24.3)	0.81 (3.1)	42 (7.3)	5.73 (3.12, 10.42)	30 (11.6)	2.15 (4.13)	10 (5.4)	6.78 (2.08, 10.42)
Bicycle	552 (33.3)	0.73 (2.85)	70 (12.1)	3.12 (2.08, 7.29)	37 (14.2)	3.21 (6.38)	15 (8.1)	5.21 (2.08, 10.42)
Ox cart/wheelbarrow	150 (9.1)	1.43 (5.45)	22 (3.8)	9.37 (2.08, 10.42)	1 (0.4)	10.42 (.)	1 (0.5)	10.42 (10.42, 10.42)
Car/Taxi/Bus	455 (27.5)	7.33 (7.30)	381 (83.7)	6.25 (4.17, 10.42)	161 (62.3)	9.15 (9.43)	142 (76.8)	7.81, (4.17, 12.50)
Motorcycle	88 (5.3)	4.98 (6.91)	61 (10.5)	5.21 (3.12, 9.37)	14 (5.4)	10.08 (9.24)	11 (5.9)	10.42 (6.25, 20.83)
Ambulance	8 (0.5)	3.25 (6.19)	2 (0.3)	13.02 (10.42, 15.62)	16 (6.2)	3.74 (6.11)	6 (3.2)	9.37 (7.81, 10.42)
Spent on transport regardless of method^a^	-	-	589 (35.3)	6.25 (3.12, 10.42)	-	-	185 (71.1)	7.81 (4.17, 12.50)

**Notes:** ^‡^The following were excluded from the analysis: 77 (3.2%) women who delivered on the way to a health facility, 17 (0.7%) women whose spending behaviors were considered extreme outliers, 6 (0.3%) women who were missing a delivery location, and 1 (0.04%) woman who lived too close to her assigned primary health center. ^a^Proportion of women who delivered at a primary health center or hospital and reported spending on transportation, regardless of the method they used.

Qualitatively, respondents frequently discussed women who delivered at home discussed needing transport to and from the health center immediately after delivery for postnatal checks or for transport if the woman delivered at a home other than her own:

“The person who has delivered at home … has to use more money to go to the clinic after delivery and for other things.” – Woman, Lundazi District (delivered at a primary health center)

Accommodation, provider fees, and other expenses remained minimal regardless of delivery location, with some large outliers noted (Figure S1). Qualitatively, women reported facing monetary or in-kind charges levied by the health center or local traditional leadership for home deliveries. These fees were frequently discussed during IDIs by a majority of respondents, regardless of their actual delivery location, as a reason to not deliver at home:

“(For home birth) you are charged. You need to take a goat to the headman. Then at the clinic, you take 50 kwacha (USD5.21).” – Woman, Lundazi District (delivered at home)

“If you end up delivering in an ox cart when you’re going there (to the health center), you have to pay.” – Woman, Mansa District (delivered at home)

“If I had delivered at home, I would have been charged 200 kwacha (USD20.83) and 5 kwacha (USD0.52) for the growth monitoring card (at the health center).” – Woman, Choma District (delivered at primary health center)

However, quantitatively, only 3.9% of women who delivered at home reported spending on health center fees (excluding medicines and diagnostic tests), and 5% on informal and inkind payments (excluding tips). Women reported spending a median of USD1.56 on either category (data not shown).

### Associations between demographics and expenditure

Among women who reported any expenditure, women with no education had over seven times the odds (p<0.001) of spending anything and spent USD1.46 (p=0.022) less on their delivery compared to women with any primary education, while women with more than primary education had the same odds of spending but spent USD2.16 (p<0.001) more after controlling for all other predictors in the model (Table 3). Similarly women in the lowest wealth quartile spent USD1.61 less (p=0.002) on delivery compared to women in the second wealth quartile, while women in the two highest quartiles spent more (by USD1.41 and USD3.14, respectively; p=0.010, p<0.001). Women who did not save for delivery spent USD3.35 less (p<0.001) than those who did, while women who delivered in the dry season spent nearly USD3.88 less (p<0.001) compared to rainy season deliveries. All districts spent considerably more compared to Lundazi District, with Manse/Chembe spending USD13.32 more (p<0.001) after controlling for all other predictors in the model.

**Table 3:** Predictors of any expenditure (Total Expenditure>0) for delivery and a linear regression of the natural log of total expenditure by women living ≥10 km from their assigned primary health center who also spent money on their delivery^‡^

		Odds ratio for any expenditure (total expenditure>0)		Natural log of total expenditure amount			
Variable		Adjusted odds ratios^a^	p-value	Adjusted coefficent (95% CI)^b^	p-value	Adjusted effect (USD) (95% CI)^b^	p-value
**Women’s Age**	15–19	1.77 (0.59, 5.24)	0.305	0.00 (−0.07, 0.08)	0.953	0.00 (−1.14, 1.41)	0.953
	20–24	1.00		1.00		1.00	
	25–29	0.83 (0.35, 1.95)	0.660	0.06 (−0.01, 0.13)	0.082	1.05 (−0.17, 2.35)	0.082
	30–34	0.64 (0.23, 1.77)	0.388	0.07 (0.00, 0.13)	0.048	1.23 (0.00, 2.35)	0.048
	35+	0.88 (0.30, 2.59)	0.811	0.05 (−0.03, 0.12)	0.212	0.87 (−0.50, 2.16)	0.212
**Education**	None	7.04 (2.28, 21.77)	<0.001	−0.09 (−0.17, −0.01)	0.022	−1.46 (- 2.65, - 0.17)	0.022
	Any primary	1.00		1.00		1.00	
	More than Primary	1.32 (0.57, 3.15)	0.499	0.12 (0.08, 0.19)	<0.001	2.16 (1.41, 3.54)	<0.001
**Marital Status**	Never married	0.48 (0.18, 1.30)	0.505	−0.05 (−0.14, 0.05)	0.308	−0.83 (−2.21, 0.87)	0.308
	Divorced/seperated/widowed	0.54 (0.18, 1.62)	0.554	−0.03 (−0.12, 0.05)	0.416	−0.50 (- 1.92, 0.87)	0.416
	Married/cohabiting	1.00		1.00		1.00	
**Wealth Index**	1 (lowest)	0.54 (0.20, 1.45)	0.221	−0.10 (−0.17, −0.03)	0.002	−1.61 (−2.65, −0.50)	0.002
	2	1.00		1.00		1.00	
	3	1.07 (0.45, 2.61)	0.887	0.08 (0.02, 0.14)	0.010	1.41 (0.34, 2.55)	0.010
	4 (highest)	1.89 (0.66, 5.41)	0.232	0.17 (0.10, 0.24)	<0.001	3.14 (1.78, 4.60)	<0.001
**District**	Kalomo	0.44 (0.15, 1.33)	0.145	0.21 (0.12, 0.30)	<0.001	3.96 (2.16, 5.93)	<0.001
	Choma/Pemba	0.68 (0.24, 1.93)	0.465	0.29 (0.21, 0.37)	<0.001	5.70 (3.96, 7.59)	<0.001
	Lundazi	1.00		1.00		1.00	
	Nyimba	0.23 (0.07, 0.70)	0.010	0.42 (0.33, 0.52)	<0.001	8.84 (6.62, 11.56)	<0.001
	Mansa/Chembe	0.97 (0.24, 3.87)	0.968	0.58 (0.49, 0.67)	<0.001	13.32 (10.71, 16.17)	<0.001
**Primigravida**	No	1.00		1.00		1.00	
	Yes	0.54 (0.19, 1.53)	0.244	0.07 (0.00, 0.14)	0.048	1.23 (0.00, 2.55)	0.048
**Four or more ANC visits**	No	0.42 (0.20, 0.87)	0.020	−0.04 (−0.09, 0.01)	0.134	−0.66 (−1.46, 0.17)	0.134
	Yes	1.00		1.00		1.00	
**Saved for delivery**	No	0.11 (0.06, 0.23)	<0.001	−0.22 (−0.29, −0.16)	<0.001	−3.35 (−4.26, −2.50)	<0.001
	Yes	1.00		1.00		1.00	
**Season of delivery**	Rainy	1.00		1.00		1.00	
	Dry	0.53 (0.28, 1.01)	0.054	−0.26 (−0.33, −0.19)	<0.001	−3.88 (−4.73, −2.93)	<0.001
**Distance from health center**	9.5–10	0.55 (0.17, 1.76)	0.315	−0.07 (−0.17, 0.03)	0.194	−1.14 (−2.65, 0.52)	0.194
10–14.9	1.00		1.00		1.00	
	15–19.9	1.19 (0.44, 3.27)	0.729	0.00 (−0.08, 0.08)	0.985	0.00 (−1.30, 1.41)	0.985
	20–24.9	1.90 (0.68, 5.27)	0.218	0.00 (−0.08, 0.08)	0.999	0.00 (−1.30, 1.41)	0.999
	25+	1.02 (0.27, 3.80)	0.976	0.02 (−0.07, 0.12)	0.885	0.34 (−1.14, 2.16)	0.885
				Intercept:	2.83		
				p*-*value:	<0.001		
							

**Notes:** ^‡^The following were excluded from the analysis: 77 (3.2%) women who delivered on the way to a health facility, 17 (0.7%) women whose spending behaviors were considered extreme outliers, 6 (0.3%) women who were missing a delivery location, and 1 (0.04%) woman who lived too close to her assigned primary health center. ^a^Adjusted for all variables shown in Table 3. ^b^Adjusted for all variables shown in Table 3 and delivery location (Table 4)

**Table 4:** Two-part model of expenditures for recently delivered women living ≥10 km from their assigned primary health center^‡,a^

		**Total Expenditure for Delivery**						
		**Logistic Regression**		**Natural Log**				
		**Adjusted odds ratios (95% CI)**	**p-value**	**N**	**Adjusted coefficient** **(95% CI)**	**p-value**	**Adjusted effect (USD)** **(95% CI)**	**p-value**
**Delivery Location**	Home	1.0		332	1.0		1.0	
	Primary health center	4.13 (2.07, 8.24)	<0.001	1635	0.21 (0.14, 0.28)	<0.001	3.96 (2.55, 5.47)	<0.001
Hospital	6.14 (1.74, 21.63)	0.005	256	0.37 (0.28, 0.46)	<0.001	7.59 (5.47, 9.90)	<0.001
**Intercept**					2.83	<0.001		
		**Expenditure on Baby Clothes/Baby Blanket**						
		**Logistic regression**		**Natural Log**				
		Adjusted odds ratios (95% CI)	p-value	N	Adjusted coefficient (95% CI)	p-value	Adjusted effect (USD) (95% CI)	p-value
**Delivery Location**	Home	1.0		319	1.0		1.0	
	Primary health center	3.90 (2.30, 6.64)	<0.001	1616	0.14 (0.08, 0.21)	<0.001	2.02 (1.12, 3.15)	<0.001
Hospital	3.01 (1.18, 7.69)	0.021	252	0.18 (0.08, 0.27)	<0.001	2.65 (1.12, 4.17)	<0.001
**Intercept**					2.60	<0.001		
		**Expenditure on Delivery Supplies**						
		**Logistic Regression**		**Natural Log**				
		Adjusted odds ratios (95% CI)	p-value	N	Adjusted coefficient (95% CI)	p-value	Adjusted effect (USD) (95% CI)	p-value
**Delivery Location**	Home	1.0		235	1.0		1.0	
	Primary health center	2.91 (2.16, 3.92)	<0.001	1424	0.02 (−0.11, 0.15)	0.748	1.05 (−0.49, 0.77)	0.748
Hospital	3.45 (2.12, 5.63)	<0.001	219	0.27 (0.08, 0.46)	0.005	1.47 (0.40, 2.78)	0.005
**Intercept**					1.56	<0.001		
		**Expenditure on Transportation**						
		**Logistic Regression**		**Natural Log**				
		Adjusted odds ratios (95% CI)	p-value	N	Adjusted coefficient (95% CI)	p-value	Adjusted effect (USD) (95% CI)	p-value
		**Expenditure on Transportation**						
		**Logistic Regression**		**Natural Log**				
		**Adjusted odds ratios (95% CI)**	**p-value**	**N**	**Adjusted coefficient** **(95% CI)**	**p-value**	**Adjusted effect (USD)** **(95% CI)**	**p-value**
**Delivery Location**	Home	1.0		43	1.0		1.0	
	Primary health center	4.42 (2.94, 6.63)	<0.001	581	−0.14 (−0.36, 0.07)	0.197	−0.86 (−1.98, 0.47)	0.197
Hospital	16.87 (9.76, 29.16)	<0.001	185	0.02 (−0.18, 0.23)	0.806	0.13 (−1.08, 1.69)	0.806
**Intercept**					1.88	<0.001		

**Notes:** ^‡^The following were excluded from the analysis: 77 (3.2%) women who delivered on the way to a health facility, 17 (0.7%) women whose spending behaviors were considered extreme outliers, 6 (0.3%) women who were missing a delivery location, and 1 (0.04%) woman who lived too close to her assigned primary health center. ^a^Controlled for the variables in Table 3.

Marital status, four or more ANC visits, and village distance from health center were not significantly associated with total expenditure. Similar results were found when exploring associations between covariates and expenditure on baby clothes, delivery supplies, and transport (Table S1).

### Associations between delivery location and expenditure

Compared to women who delivered at home, women who delivered at a primary health center had over four times the odds (p<0.001) of spending anything on their delivery and spent approximately USD4 (p<0.001) more for their delivery in total and USD2 (p<0.001) more specifically for baby clothes/a baby blanket, after adjusting for all other predictors (Table 4). Women who delivered at a primary health center also had nearly three times the odds (p<0.001) of spending on delivery supplies and over four times the odds of spending on transportation (p<0.001), yet spent about the same amount on either of these categories as women who delivered at home.

Hospital deliveries, as expected, were more costly, since women delivering at a hospital had over six times the odds of spending anything on delivery compared to women who delivered at home, and spent USD7.59 (p<0.001) more in total, over USD2.50 (p<0.001) more for baby clothes/baby blanket, and nearly USD1.50 more for delivery supplies (p=0.005). Women who delivered at a hospital were nearly 17 times as likely to spend on transporation but did not spend more on that category when compared to women who delivered at home.

## Discussion

Though facility user fees for maternal health care services in Zambia were abolished in 2006,^18^,^32^ expenditure for maternity services remains a frequently cited barrier to facility-based delivery.^21^,^22^,^33^–^36^ We conducted a cross-sectional, mixed-methods study with the most rural Zambian women to determine how much women are paying for delivery and to assess how they experience these delivery expenses. With our sampling methodology, we have not only reached some of the most rural, but also some of the poorest women in Zambia, who are most likely to be hindered from accessing timely and quality maternity care due to its associated costs. Any statistically significant differences in the demographics of women who reported spending with those who reported zero spending for delivery are not programmatically meaningful.

### Total expenditure

Mean total delivery expenditure was approximately USD29 among all women sampled, regardless of delivery location, higher than what has been reported in similar, user fee free settings, including Tanzania (approximately USD5);^37^,^38^ Burkina Faso (approximately USD7);^38^ and Kenya (approximately USD14)^38^ for normal or complicated deliveries occurring at government-run or private health facilities and hospitals. As the data for these studies were collected over a decade ago, the comparison may not be as relevant. However, there is extremely limited contemporary information on household OOP expenditure in sub-Saharan Africa for maternity health services.

As expected, delivery location affects how much women spend, though not to the degree we anticipated. It is least expensive for rural Zambian women to deliver at home (mean USD21.82) and most expensive for them to deliver at a hospital (mean USD36.46), with primary health center-based deliveries falling in between (mean USD29.07). Unexpectedly, household expenditure for home deliveries was much higher in our study than the study by Perkins et al, which showed that expenditure for home delivery was USD0.4, USD1, and USD3.6 in Burkina Faso, Tanzania, and Kenya, respectively.^38^

Considering that the average monthly household income for the poorest households in Zambia is approximately USD105,^39^ these delivery expenditures, regardless of delivery location, account for roughly one-third of a household’s monthly income. Furthermore, rural Zambian households already spend nearly 60% of their monthly income on food,^39^ meaning delivery expenses amount to nearly all of the remaining monthly income. This is a substantial amount of expenditure regardless of whether a household spends all of it in one month or is able to save and plan for delivery, speading the cost over several months. Household savings built up during pregnancy may be important for affording these costs.^40^

### Baby clothes: a surprising driver of expenditure

The baby clothes category which includes baby clothes themselves and a baby blanket make up over 75% of delivery expenses, and nearly all (95.9%) women reported spending on this category. While previous qualitative studies have discussed baby clothes as a perceived obstacle to facility delivery among Zambian households,^11^,^21^,^22^,^33^–^35^,^41^ it is a novel finding that these baby clothes make up such a high proportion of total reported expenditure among the most rural women. Corroborating this, qualitatively, rural Zambian women report feeling substantial pressure from health center staff to bring new baby clothes with them to delivery and report feeling shamed if they do not, which confirms and elaborates previous findings.^11^,^33^,^34^ Previous studies which reported much lower total delivery expenditures elsewhere in sub-Saharan Africa may have omitted this category of expenditure. Further studies in other countries in the region could help determine the scope of these unofficial requirements, and how they relate to societal norms and expectations.

### Delivery supplies: not the driver we anticipated

The perceived need to bring delivery supplies for a facility-based delivery remains an important barrier for rural Zambian women. Nearly all (82.4%) women in this study, regardless of delivery location, reported purchasing delivery supplies in preparation for their delivery. The need to procure supplies for delivery, including disinfectant, cord clamps, and a razor blade, has been explored in other studies^11^,^22^,^34^,^35^,^41^ and was cited as potentially a main reason that facility-based deliveries did not increase after the abolition of user fees.^20^ While the abolition of user fees may have shifted the burden for resource mobilization from the health system to the user, the need to bring supplies for a facility-based delivery is not a new phenomenon in rural Zambia,^35^ though user fee removal may have expanded the practice.

Since delivery supplies were mentioned frequently in our qualitative data and in previous literature,^11^,^20^,^22^,^34^,^35^,^41^ we anticipated that the reported expenditure for these supplies would be much higher than the mean amount of approximately USD4, or about 13% of total expenditures. This is slightly less than the low end of reported delivery supplies expenditure in other countries where women spend USD5 to USD14.^37^,^38^ It is possible that the effort to procure the supplies – having to travel into town and purchase items at different shops after identifying which shops have the supplies in stock – could be the greater obstacle, due to transport and opportunity costs (not captured in this study), than the cost of the items themselves.

Surprisingly, women who delivered at home spent almost as much on delivery supplies, with women who delivered at a primary health center or hospital spending only USD1 and USD1.50 more after controlling for all other factors. These amounts are minimal compared to the amounts spent on baby clothes discussed above.

### Transportation: not in line with other studies

Previous studies in Zambia have highlighted the cost and availability of transport as an important barrier to facility-based delivery.^21^–^23^,^34^,^35^,^42^ Yet, transportation expenditure was lower than expected based on our household survey results. Among our sample of the most rural Zambian woman, women who delivered at a primary health center or hospital were almost four and 16 times as likely, respectively, to spend anything on transportation compared to women who delivered at home. Yet, median expenditure for transport was USD6.25 (compared to USD26 for median total expenditure) for either home or primary health center deliveries, increasing slightly (USD7.81) for a hospital delivery. Although, transportation method affected transport expense as expected, the qualitative responses did not corroborate this as much as we would have expected. As with delivery supplies, the stress, effort, and uncertainty of coordinating and obtaining transportation may be a greater barrier than the financial expense, especially when a woman’s labor has already begun.

### Penalties for home deliveries

There is a widely held belief throughout rural Zambia that women who deliver either at home or before arriving to a health facility will be penalized by having to pay for the child’s otherwise free “under-5 card” (for growth monitoring and vaccination documentation) or they will be charged by the local traditional leadership, often in the form of livestock. While this was frequently discussed qualitatively as a deterent for home delivery, and has been previously reported on,^43^ few women reported paying such fees either in cash or in-kind in our study. Either this practice has waned substantially since data were initially collected about it in 2012/2013, the practice was never as widespread as previously believed, or women did not report such penalties in our household survey. Regardless of how widespread the actual practice may be, fear of penalties remains an important deterrant to home births, but continues to raise ethical concerns.^43^

### Recommendations

We have provided specific recommendations for the Ministry of Health and local implementing organizations in Zambia in Box 1, relevant to each of the major expenditure categories.

### Limitations

This study had several limitations. First, the kwacha-dollar exchange rate changed considerably over the time period when women in this study were purchasing delivery items, with the kwacha falling in value against the dollar. While it is not likely that the costs of items changed as quickly as the exchange rate, especially in the most rural areas in Zambia, reporting the results in US dollar may make the costs appear lower than they are experienced on the ground. Second, the data presented here are specific to the most rural populations in three provinces in Zambia and may not reflect the delivery expenses incurred by the average Zambian woman.

Regarding baby clothes and delivery supplies, the household survey did not specifically ask respondents about the transport expense or opportunity costs associated with procuring those items, so their true cost may be higher than presented. We did not capture where women are purchasing baby clothes and delivery supplies, which potentially could affect both their costs and shed light on logistical challenges to procurement. Furthermore, while questions in the household survey asked for roundtrip transport expenses to and from the location of delivery, only the main method of transport used to get to the location was included in the questions. Women may have utilized different methods of transport during their roundtrip that influenced their total transport costs.

When asking about delivery location, we did not distinguish between public, private, or mission facilities, so we were unable to disaggregate the analysis. Some women may have incurred higher costs by visiting private facilities. However, in the context of rural Zambia, there are limited options for private facilities, so it is unlikely any more than a very small proportion of our sample would have utilized a private facility. Mission facilities follow the same user fee free policies as government-run centers, though may have additional financial resources from outside sources. Lastly, we were unable to distinguish between complicated and uncomplicated deliveries.

## Conclusion

While Zambia has abolished official user fees for maternal health services, our findings make clear there is no such thing as a free delivery. The reduction of delivery-associated expenses incurred by women must be addressed, otherwise the poorest and most vulnerable women in Zambia will continue to face financial barriers to accessing adequate obstetric care.

**Box 1:** Recommendations based on study findings

While the abolition of user fees has reduced the provider fees for delivering at a facility, poor, rural Zambian women still face high financial expenditures related to delivery, a problem that may impede Zambia’s efforts to achieve universal health coverage. Understanding the actual OOP delivery expenditure for these remote women is important to target interventions trying to expand facility-based delivery and improve maternal and newborn health outcomes. These interventions can target either the health system, communities, or both.
**1) Baby Clothes/Blanket** The unofficial requirements or norms for bringing new baby clothes and a baby blanket to a facility delivery in Zambia results in the largest expense for nearly all remote pregnant women in this study, which is likely a widespread phenomenon across the country as it has been found qualitatively in other studies.^11^,^33^,^44^ Blankets should be recognized as the underlying driving expense for this category. The Zambian Ministry of Health could consider examining the unofficial policy of bringing new baby clothes/blanket to a facility-based delivery and its implications. However, as this expectation has become so associated with social embarrassment, the norm may be hard to change. A way around this norm may be for health staff to advise women to bring clean clothing/blanket, instead of specifically new clothing/blanket, to a facility-based delivery. To address the high cost of baby clothes and baby blankets and mitigate it as a barrier, future programs could strategize on how to make these clothes and blankets more physically and financially accessible such as by promoting social enterprises to bring down their cost and extend the availability of lower priced clothing/blankets to rural areas. Furthermore, village savings groups and birth preparedness interventions^40^,^45^,^46^,^47^,^48^ could assist women in rallying the sufficient resources to more easily purchase these items.
**2) Delivery Supplies** Though minimal in comparison to total expenditure, the shifted burden for acquiring delivery supplies nonetheless remains a substantial perceived burden to accessing facility-based delivery. The Zambian Ministry of Health should consider health systems strategies to ensure health facilities have adequate financial resources to provide supplies of disinfectant, cord clamps, a razor blade to cut the cord, plastic sheets, and buckets to receive the placenta to every laboring woman free of charge.
**3) Transport** Lastly, our study suggests that the availability of transport and physical obstacles may be a more important barrier to health facility delivery than the cost of transport itself, though this is difficult to untangle. Interventions to increase physical access to health facilities for delivery could focus on improving planning for transport or providing residential space for women to wait for delivery near health facilities, known internationally as maternity waiting homes.^25^,^34^,^36^,^44^,^49^,^50^

## Data Availability

The authors will provide the de-identified household survey and in-depth interview demographic data upon reasonable request to the Principle Investigator, Dr. Nancy A Scott, at nscott@bu.edu. The in-depth interview transcripts are not publicly available due to ethical restrictions on publicly sharing data which are of sensitive nature and contain potentially identifiable information instituted by the Boston University IRB and the ERES Converge IRB in Zambia. Qualitative data requests may be sent to the Boston University IRB at medirb@bu.edu.

## Abbreviations list

ANC, antenatal care; BEmONC, basic emergency obstetric and neonatal care; CI, confidence interval; IDI, In-depth interview; IRB, institutional review board; KM, kilometer; MWK, Malawian kwacha; OOP, Out-of-pocket; USD, United States dollar; ZMW, Zambian kwacha.
